# A Nurse's View of Hospital Patients

**Published:** 1887-10-15

**Authors:** 


### A NURSE'S VIEW OF HOSPITAL PATIENTS.

As a nurse of many years' standing, whose experience has been chiefly gained in metropolitan hospitals, would you allow me to make a few observations on the letter of a Country Surgeon? First as to friends. In most, if not all London hospitals, Sunday afternoon is "visiting time," and surely no time more convenient to working-men could be fixed on. In cases of emergency, permission to visit a patient can always be obtained from the house-surgeon, matron, or ward-sister. In cases of dangerous illness, friends are admitted at all times, and can, if they wish it, remain at the bedside during the night. As to diet, five meals a day are provided, viz., breakfast, luncheon, dinner, tea, and supper. Besides which, restless or feverish patients are given milk, wine, or lemonade, etc., through the night. I have always found 11 a.m. a most busy time in a hospital ward, and I am afraid, if the nurses were to devote themselves to the concocting of "possets" according to the fancy of each patient, matters even of more importance would be neglected. I have often thought that there might with advantage be more variety in hospital diet; but as the steward of a large hospital may have many more difficulties to contend with than the matron of a cottage hospital, I do not like to speak with certainty on this subject, except to mention that milk pudding daily is included in all diets not strictly fluid. As to tea, butter, and sugar, I have had experience of hospitals where those articles were and were not provided, and decidedly prefer the latter system. Unless they are provided of the best quality, and of unlimited quantity (especially the butter), the patients always like best to find their own. Very few are absolutely unable to do so, and when they are, there is always some way, too long to mention here, of getting out of the difficulty. I have never known a patient reduced to going without. I am afraid a Country Surgeon must have got his information from one of those inveterate grumblers who are occasionally to be found in every hospital ward.—Yours faithfully, W. S.

General Infirmary, Hertford, Oct. 10, 1887.

